# Energy Homeostasis and Body Weight before and after Cessation of Block and Replacement Therapy in Euthyroid Patients with Graves' Disease

**DOI:** 10.1155/2011/715370

**Published:** 2011-11-29

**Authors:** Lars P. Klieverik, Andries Kalsbeek, Mariëtte T. Ackermans, Hans P. Sauerwein, Wilmar M. Wiersinga, Eric Fliers

**Affiliations:** ^1^Department of Endocrinology and Metabolism, Academic Medical Center F5-162, University of Amsterdam, Meibergdreef 9, 1105 AZ Amsterdam, The Netherlands; ^2^Department of Clinical Chemistry, Laboratory of Endocrinology, Academic Medical Center, University of Amsterdam, Amsterdam, The Netherlands

## Abstract

Patients with Graves' hyperthyroidism (GH) treated with a combination of thyrostatic drugs and T_4_, that is, block and replacement therapy (BRT), often report body weight (BW) gain. We aimed to determine changes in BW and energy metabolism upon cessation of BRT in these patients, and to identify possible endocrine determinants. We analysed 22 patients with GH (i) during BRT, and (ii) 12 weeks after BRT cessation. Patients were euthyroid at both visits. There were no differences in BW or resting energy expenditure (REE) between visits. At visit 1, after 13.5 (9.5–48.0) months of BRT, serum free (F)T_4_ correlated positively with REE (*r* = 0.433, *P* = 0.044) and negatively with body fat % (*r* = -0.450, *P* = 0.035). Plasma FT_3_ and FT_3_/FT_4_ ratio showed an increase 12 w after cessation of BRT (20%, *P* < 0.0001 and 16%, *P* = 0.007, resp.). Moreover, the relative change in FT_3_/FT_4_ ratio showed a significant, positive correlation with the relative change in REE between the 2 visits (*r* = 0.465, *P* = 0.029). In conclusion, serum FT_4_ determines REE in euthyroid patients with GH treated with BRT. Twelve weeks after BRT cessation, BW and energy homeostasis are unaltered. However, as serum FT_3_/FT_4_ ratio increases after cessation of BRT, which is a positive determinant of changes in REE, a longer term BW decrease is likely to occur.

## 1. Introduction

Patients with a first episode of Graves' hyperthyroidism (GH) are often treated initially with pharmacological therapy. Many clinicians use a therapeutic regimen commonly referred to as “block and replacement therapy” (BRT) to attain biochemical euthyroidism. This involves the administration of a thyroid-hormone- (TH-) synthesis-blocking agent such as methimazole (MMI) or propylthiouracil (PTU) to which L-thyroxine (T_4_) is added once serum TH concentrations reach the euthyroid range. In other causes of hyperthyroidism, such as toxic multinodular goiter or in severe cases of GH, radio-iodine ablation or (partial) surgical thyroidectomy may be used as an initial means to block thyroid hormone secretion, often combined with T_4_ supplementation at a later stage. 

Hyperthyroidism is associated with profound changes in energy homeostasis that usually resolve upon treatment with BRT. As a result, the weight loss experienced during hyperthyroidism is usually regained during treatment. Some older studies in hyperthyroid subjects have reported no difference between premorbid body weight (BW) and BW after one year of treatment [1, 2], fitting with the notion of tight BW set-point regulation. However, a more recent study in 162 patients reported a continuing BW gain after treatment for hyperthyroidism. BW had increased by ~4 kg after 1 year and increased by up to 10 kg after four years of treatment [3]. In line with this observation, up to 84% of patients report weight gain exceeding their premorbid BW following treatment of hyperthyroidism [4]. Although the etiology of this excessive weight gain is incompletely understood at present, some authors have proposed that it may result from subnormal energy expenditure due to iatrogenic suppression of TH concentrations to the lower end of the normal range [1, 5]. 

It is remarkable that to date studies addressing this issue have focussed on the comparison between BW and energy homeostasis in untreated hyperthyroid patients with the euthyroid situation during BRT. Approximately 50% of patients with Graves hyperthyroidism remain euthyroid following cessation of BRT after at least one year of treatment, reflecting the remission rate of autoimmune hyperthyroidism [6]. In our outpatient clinic of endocrinology, BRT is discontinued in patients with Graves hyperthyroidism after approximately one year of treatment. This regimen offers a good opportunity to investigate changes in BW upon cessation of BRT in euthyroid patients with Graves disease and to identify determinants of BW and energy metabolism in this setting.

## 2. Materials and Methods

### 2.1. Subjects

Patients were recruited from the Outpatient Clinics of Endocrinology and Metabolism at the Academic Medical Center of the University of Amsterdam. Inclusion criteria were (i) a minimum of six months of MMI or PTU in combination with T_4_ pharmacotherapy for Graves' hyperthyroidism, diagnosed on the basis of standard biochemical and scintigraphic criteria [7], (ii) biochemical euthyroidism (defined as serum free T_4_ (FT_4_) between 9 and 23 pmol/L, serum T_3_ between 1.3 and 2.7 nmol/L and serum TSH <5 mU/L) on both study occasions, and (iii) age between 20 and 60 years. As low or even suppressed serum TSH values can be found in euthyroid patients treated for Graves hyperthyroidism, probably resulting from binding of circulating TSH receptor (TSH-R) autoantibodies (TBII) to the TSH-R on the thyrotrophs [8], patients with serum TSH values below the lower limit of the reference range could participate in the study. 

Exclusion criteria were (i) pregnancy, (ii) abnormal liver function as apparent from serum alanine-aminotransferase >45 U/L, aspartate-aminotransferase >40 U/L or gamma-glutamyl-transferase >60 U/L, and (iii) abnormal kidney function (serum creatinine >95 *μ*mol/L) at inclusion. 

The study was approved by the Medical Ethical Committee of the Academic Medical Center of the University of Amsterdam and accordingly, written informed consent was obtained from all subjects prior to inclusion.

### 2.2. Protocol

In this observational study, the participants were studied in the morning after an overnight fast on two occasions, that is, (i) during biochemical euthyroidism while on BRT (visit 1) and (ii) during biochemical euthyroidism, 12 weeks after discontinuation of BRT (indicating remission of Graves' hyperthyroidism) (visit 2). At visit 1, a medical history was obtained regarding premorbid BW, initial presentation of Graves hyperthyroidism. Data on BW progression during the course of treatment were obtained from clinic records for every individual patient. On both occasions, BW, resting energy expenditure (REE), body composition, and serum T_3_, T_4_, FT_3_, FT_4_, reverse T_3_ (rT_3_), TSH, insulin, glucose, adrenalin, and noradrenalin were assessed. All measurements were performed in the morning between 8.00 and 12.00 AM. Of all patients that were included in the study, one patient used a *β*-adrenergic blocker (metoprolol), and one patient used a benzodiazepine (diazepam). Both patients were instructed not to take any co-medication within at least 24 h prior to study measurements. All patients were instructed not to take thyroxine in the morning of visit 1, and smoking was not allowed within 12 h prior to measurements. Patients were instructed to prevent physical exercise at least 3 days before both study visits, to eat 3 meals per day, and not to change their eating or exercise habits between the visits. Importantly, patients were instructed to refrain from any specific diet during the study period.

### 2.3. Body Composition and Indirect Calorimetry

Body composition was measured using bioelectrical impedance analysis (Maltron BF-906, Rayleigh, UK). Oxygen consumption (*V*O_2_) and CO_2_ production (*V*CO_2_) were measured with 20 sec intervals for a total time of 30 min by indirect calorimetry using a ventilated hood system (Sensormedicsmodel 2900; Sensormedics, Anaheim, Calif, USA). Patients were studied in the morning after an overnight fast. During and 30 min prior to the calorimetry measurements, subjects were instructed to rest in the supine position in a temperature-controlled room (23°C). REE, lipid, and glucose oxidation were calculated from *V*O_2_ and* V*CO_2_ values using algorithms previously reported by Frayn [9]. Respiratory quotient (RQ) was calculated as* V*CO_2_/*V*O_2_. The final 25 min of each calorimetry measurement, during which stable *V*O_2_ and* V*CO_2_ values are reached, was averaged for further analysis in every individual patient.

### 2.4. Hormones

Serum T_3_ (total), T_4_ (total), and rT3 were measured with in-house radioimmunoassay (RIA) [10]. Serum FT_4_, FT_3_, and TSH were measured by time-resolved fluoroimmunoassay (Delfia, Wallac Oy, Turku, Finland). For FT_4_ the intra-assay variation was 4-5%, the inter-assay variation 6-7%, and the detection limit 2 pmol/L. For FT_3_ the intra-assay variation was ±6%, the inter-assay variation ±9%, and the detection limit 1 pmol/L. For TSH the intra-assay variation was 3-4%, the inter-assay variation 4-5%, and the detection limit 0.01 mU/L. Insulin was measured with a chemiluminescent immunometric assay (Immulite 2000 system, Diagnostic Products Corporation, Los Angeles, Calif, USA) with an intra-assay variation of 3–6%, an inter-assay variation of 4–6%, and a detection limit of 15 pmol/L. Serum TBII was quantitatively determined by a second-generation luminescence receptor assay (DYNOtest TRAK human assay, B.R.A.H.M.S.). Noradrenalin and adrenalin were determined with an in-house HPLC method. Intra-assay variation for noradrenalin: 2% and adrenalin 9%; inter-assay variation for noradrenalin: 10% and adrenalin: 14–18%; detection limit was 0.05 nmol/L for both hormones.

### 2.5. Statistics

All data were analyzed using nonparametric tests. We performed comparisons between study occasions with the Wilcoxon Signed Rank test and expressed correlations as Spearman's rank correlation coefficient (*r*). SPSS statistical software version 12.0.1 (SPSS Inc, Chicago, Ill, USA) was used for statistical analysis. Data are presented as median (minimum-maximum).

## 3. Results

Thirty-nine biochemically euthyroid patients were initially included and studied before cessation of BRT. Of these patients, nine had developed a relapse of hyperthyroidism while two patients who had undergone radio-iodine (I^131^) ablation therapy had developed hypothyroidism at visit 2. Six patients were excluded from the final analysis on the basis of serum FT_4_ or T_3_ values outside the reference range, or serum TSH values above the upper limit of the reference range. Thus, the final analysis was performed in 22 patients who were biochemically euthyroid at both visits. 

### 3.1. Anthropometric Characteristics

Baseline anthropometric characteristics are depicted in Table 1. Out of 22 patients, 2 patients (9%) smoked. These patients were instructed not to smoke 12 hours prior to measurements and not to change their smoking habits between study visits.

### 3.2. Body Weight and Energy Homeostasis

Patients reported that before the onset of the symptoms—later attributed to hyperthyroidism—that had urged them to seek medical advice, BW, that is, premorbid BW, was 64.0 (47.5–97.0) kg. Eighty-two percent (18/22) of patients reported that decreased BW was among the symptoms of hyperthyroidism. At the time of diagnosis of hyperthyroidism, the patients had lost 4.8 (0.0–25.0) kg of self-reported premorbid BW. Nineteen patients were started on methimazole, and 3 patients were started on PTU, to which thyroxine was added as soon as biochemical euthyroidism was reached. At visit 1, when BRT was discontinued, patients had been treated with BRT for 13.5 (9.5–48.0) months. During BRT patients gained 6.9 (−0.1–14.6) kg. The difference between premorbid BW and BW at visit 1 (i.e., presumed weight gain) was 2.4 (−17.1–14.6) kg (premorbid BW versus visit 1 BW, *P* = 0.001). 

There was no significant difference in median BW between visit 1 and visit 2, 12 weeks after cessation of BRT (Table 1, *P* = 0.899). The median difference in BW between visit 1 and visit 2 calculated per individual patient was 0.0 (−5.4–3.2) kg. Furthermore, there was no difference in REE, fat and glucose oxidation, RQ, or body fat mass between visit 1 and visit 2 (Table 2).

### 3.3. Hormones

**Figure 1:**
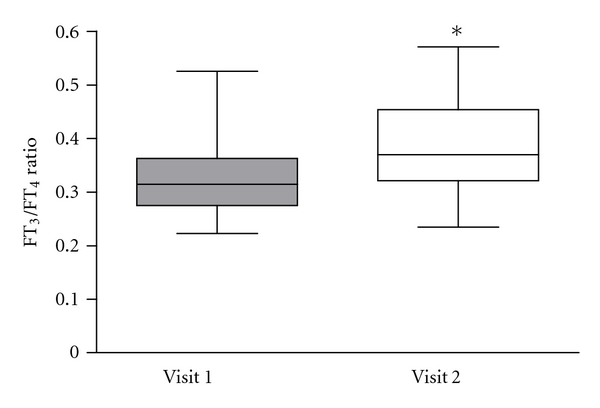
Serum FT_3_/FT_4_ ratios of 22 euthyroid Graves patients during BRT (visit 1; grey boxplots) and 12 weeks after cessation of BRT (visit 2, open boxplots). *P = 0.007, visit 1 versus visit 2.

Serum T_4_ and FT_4_ were not different between visit 1 and 2 (*P* = 0.362 and *P* = 0.676, resp., Table 3). Serum T_3_ tended to increase at visit 2 as compared with visit 1 (3%, *P* = 0.069), whereas FT_3_ showed a statistically significant increase at visit 2 (20%, *P* = 0.005). Serum TSH levels decreased by 59% and serum TBII concentrations by 15% at visit 2 compared with visit 1 (*P* = 0.001 and *P* = 0.007, resp.). Accordingly, the serum T_3_/T_4_ ratio increased by 10% from 1.79% (visit 1) to 1.97% (visit 2, *P* = 0.033), and the serum FT_3_/FT_4_ ratio increased by 16% from 31.8% (visit 1) to 37.0% (visit 2, *P* = 0.007, see Figure 1). 

**Figure 2:**
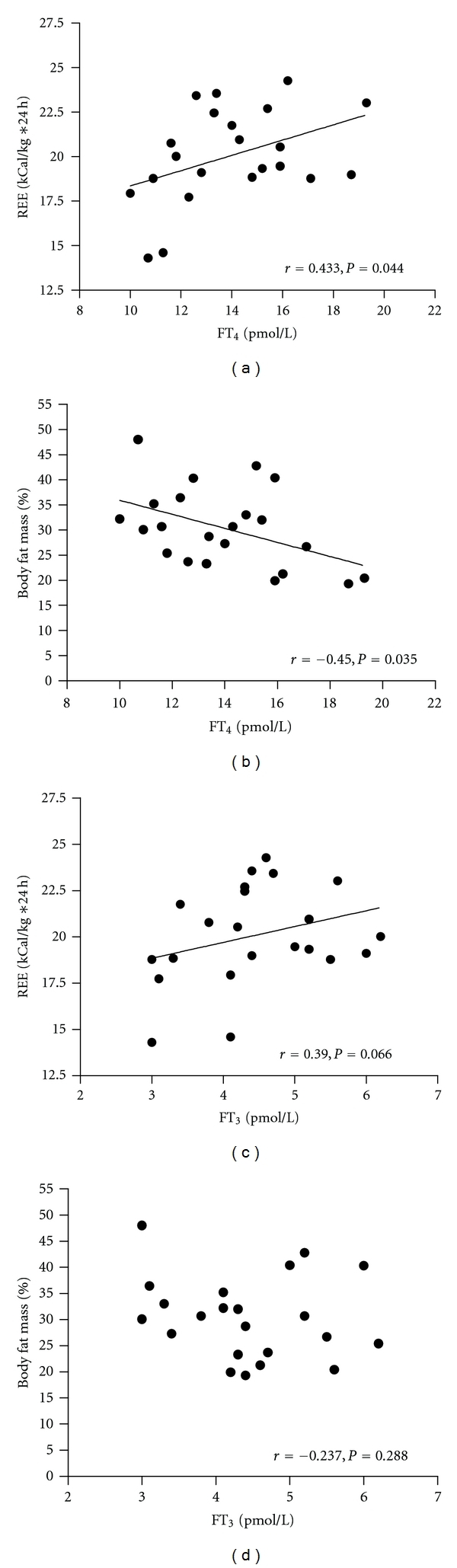
Correlations of serum FT_4_ with resting energy expenditure (REE, (a)) and body fat mass (b), and serum FT_3_ with REE (c) and body fat mass (d). Spearman's correlation coefficients (*r*) and *P* values are plotted under the horizontal axis of each graph.

At visit 1, there was a positive correlation between serum FT_4_ and REE (*P* = 0.044, Figure 2(a)) and a negative correlation between serum FT_4_ and % body fat mass (*P* = 0.035, Figure 2(b)). In addition, there was a highly significant negative correlation between REE and % body fat mass (*r* = −0.549, *P* = 0.008). Serum FT_3_ tended to correlate positively with REE (*P* = 0.066, Figure 2(c)), but showed no correlation with % body fat mass (*P* = 0.288, Figure 2(d)). 

**Figure 3:**
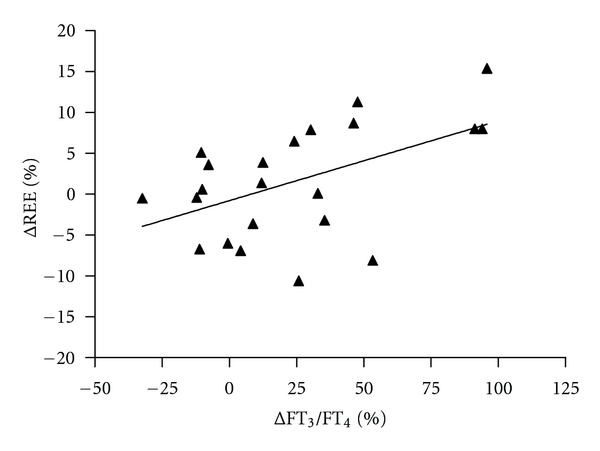
Correlation of the difference in REE corrected for BW between visit 1 and visit 2 (Δ REE (%)) and the difference in serum FT_3_/FT_4_ ratios between visit 1 and visit 2 (Δ FT_3_/FT_4_ (%)). Spearman's correlation coefficient (*r*) = 0.465, *P* = 0.029.

However, at visit 2, these significant correlations were absent (FT_4_ versus body fat %  *r* = −0.247, *P* = 0.270, FT_4_ versus REE *r* = 0.320, *P* = 0.172, FT_3_ versus body fat %  *r* = −0.109, *P* = 0.630, FT_3_ versus REE, *r* = 0.135, *P* = 0.550). Intriguingly, the difference in (Δ) FT_3_/FT_4_ ratio and ΔREE between visit 1 and visit 2 showed a positive correlation (*r* = 0.465, *P* = 0.029, see Figure 3). 

There were no differences in serum glucose, insulin, adrenalin, or noradrenalin between the two visits (Table 3).

## 4. Discussion

Patients with Graves disease who are treated with a combination of a thyreostatic drug and thyroxine replacement, that is, block and replacement therapy (BRT), often experience a marked gain in BW [3], which has been reported to exceed self-reported premorbid BW [4]. However, there are some inconsistencies in the published data on this issue. In the present study, we found a median weight excess of 2.4 kg compared with self-reported premorbid BW in a group of 22 euthyroid patients with Graves hyperthyroidism who had been treated with BRT for a median of 13.5 months. To further investigate this phenomenon we assessed energy homeostasis and serum hormone concentrations in these patients both before and 12 weeks after cessation of BRT. Somewhat unexpectedly, patients showed no change in BW or REE at 12 weeks after BRT cessation. 

With respect to the 2.4 kg BW excess, we are aware of the uncertainty regarding the reliability of self-reported premorbid BW [11]. Symptoms such as a decrease in BW are slowly progressive and patients may find it hard to remember the precise onset of symptoms. Therefore, we cannot exclude that underestimation of the self-reported premorbid BWs may have led to an overestimation of the BW excess during BRT in our patients. In any case, the present study does not support the clinical impression of a marked and excessive BW increase after ~1 year of BRT following hyperthyroidism.

Hyperthyroidism is associated with a marked increase in REE [12, 13]. The simultaneous increase in appetite and caloric intake does not completely prevent weight loss in most hyperthyroid patients [14]. By inference, BW changes appear to be primarily determined by EE in these patients. In addition, it has been shown previously that caloric intake decreases rapidly to normal within 3 months of initiation of antithyroid treatment, whereas in the same period BW increases to premorbid levels [1]. Therefore, increased caloric intake is not a likely candidate to explain the BW gain after initiation of thyreostatic treatment. Our finding that REE and BW are similar during treatment with BRT compared with 12 weeks after BRT cessation suggests that BRT does not induce a major change in energy homeostasis and, in turn, BW gain in excess of the premorbid situation. 

The patients in our study were euthyroid on both occasions, following the design of the study. This enabled us to investigate the relationship between subtle changes in serum TH values within the euthyroid range on the one hand and measures of energy homeostasis on the other hand. As anticipated, serum FT_3_/FT_4_ ratios significantly (16%) increased after cessation of BRT. This can probably be attributed to reinstated thyroidal T_3_ secretion, whereas enzymatic conversion from exogenous T_4_ is probably the major source of serum T_3_ during BRT. Interestingly, there was a significant and positive correlation between the difference in serum FT_3_/FT_4_ ratio and the difference in REE between the 2 study occasions, suggesting that the serum FT_3_/FT_4_ ratio is a positive determinant of REE under these circumstances (Figure 3). In addition, serum FT_4_ levels within the euthyroid range were a positive determinant of REE. Also FT_3_ tended to positively determine REE. This illustrates the sensitivity of REE to even small fluctuations in circulating thyroid hormone concentrations, which is supported by previous reports [15, 16]. As anticipated, REE and % body fat mass showed a highly significant negative correlation. Therefore, it is likely that the negative correlation between serum FT_4_ and % body fat mass can be explained by the observation that FT_4_ positively determines REE. It appears remarkable that these correlations reached statistical significance despite the relatively small number and heterogeneity (i.e., regarding age, sex, BMI, body fat mass) of patients. Why this was only evident during BRT treatment and not at visit 2 remains unclear. It may be that after cessation of BRT, the pathophysiological changes in the HPT-axis associated with Graves disease (e.g., by circulating TBII) prevent the detection of clear-cut associations between circulating thyroid hormones and REE. Another explanation may be that a period of 12 weeks is not sufficient to reinstate physiological thyroid hormone synthesis and release from the thyroid gland. 

Although REE and BW were unaltered 12 w after BRT cessation, the data presented do raise a number of interesting possibilities. Our finding that changes in serum FT_3_/FT_4_ ratios positively determine REE in euthyroid patients (Figure 3) suggests that tissues capable of modulating energy metabolism sense even small changes in circulating THs and alter REE accordingly. Candidates include tissues significantly contributing to REE and expressing TH receptors such as the heart and CNS, while striated muscle and adipose tissue may contribute to a lesser extent [17]. Another possibility to explain these findings may be TH-dependent regulation of REE via the brain, as the hypothalamus plays a major role in the regulation of energy metabolism [18, 19]. Interestingly, there is a high density of TH receptors in the rat and human hypothalamic arcuate and paraventricular nuclei [20, 21], and these nuclei are both key players in BW regulation [16]. Recently, we have shown that hypothalamic T_3_ administration stimulates hepatic glucose production via a neural pathway involving the hypothalamic paraventricular nucleus and the sympathetic nervous system [22]. Thus, the hypothalamus is able to sense THs and in turn modulate hepatic glucose metabolism by means of altered sympathetic outflow to the liver. This raises the possibility that it may similarly modulate energy metabolism by autonomic projections to other target tissues such as skeletal muscle and adipose tissue in response to TH [23, 24].

Serum TSH concentration was lower at visit 2 as compared with visit 1, although it remained within reference intervals. This may be explained either by subtle T_4_ under-treatment during BRT despite serum T_4_and FT_4_ levels within the reference range. Alternatively, the decrease in serum TSH may point to a tendency to develop a relapse of hyperthyroidism in some patients. However, this latter possibility appears to be less likely given the decrease of serum TBII, 12 w after BRT cessation (Table 4). 

In conclusion, we observed no changes in BW and resting energy metabolism 12 weeks after cessation of BRT in euthyroid Graves patients, so the present findings do not support the notion that BRT per se induces excessive BW gain. However, patients who stopped BRT showed an increase in the serum FT_3_/FT_4_ ratio, and these changes in FT_3_/FT_4_ ratio were a positive determinant of changes in REE. Hence, it is well possible that the changes in circulating thyroid hormones after cessation of BRT require a longer observational period in order to translate into clear-cut alterations in BW. 

## Figures and Tables

**Table 1:** Anthropometric characteristics of subjects (*n* = 22; 19 females, 4 males).

	Median	Min.–Max.
Age (yr)	45.5	24–56
Weight (kg)	67.5	49.4–106.6
Height (cm)	168	158–182
BMI (kg/ m^2^)	23.8	19.9–35.8
Lean body mass (%)	69.6	52.0–80.7
Fat mass (%)	30.4	19.3–48.0

**Table 2:** Body weight (BW).

	Median		Min.–Max.	*P*
	kg		kg	
Premorbid BW (self reported)	64.0	*a*	47.5–97.0	
BW at diagnosis GD	62.0	*b*	45.0–92.0	<0.0001 (*a* versus* b*)
BW visit 1 (during BRT)	68.1	*c*	49.4–106.6	<0.0001 (*b* versus *c*)
BW visit 2 (12 w after BRT cessation)	67.1	*d*	48.0–106.5	0.889 (*c* versus *d*)

	Median			

	kg	%		
Δ BW (visit 1–premorbid)	2.4	3.7	−17.1–14.6	
Δ BW (visit 1–visit 2)	0.0%	0.0%	−5.4–3.2	

**Table 3:** REE, substrate oxidation, and body composition.

	Visit 1		Visit 2		*P*
	Median	Min.–Max.	Median	Min.–Max.	
REE (kCal/kg∗24 h)	19.7	14.3–24.3	20.3	13.2–26.2	0.249
Body fat mass (% of total BW)	30.4	19.3–48.0	29.1	19.6–49.8	0.102
RQ	0.81	0.74–0.92	0.81	0.72–0.91	0.910
Glucose oxidation (mg/min∗kg)	1.12	0.17–2.23	1.26	0.00–2.22	0.745
Lipid oxidation (mg/min∗kg)	0.75	0.15–1.23	0.71	0.20–1.11	0.858

**Table 4:** Plasma hormone concentrations at visit 1 and visit 2.

	Visit 1		Visit 2		*P*	Reference range
	Median	Min.–max.	Median	Min.–max.		
T_4_ (nmol/L)	95	60–170	90	70–130	0.362	70–150
FT_4_ (pmol/L)	13.7	10.0–19.3	14.0	10.5–21.3	0.676	10.0–23.0
T_3_ (nmol/L)	1.68	1.2–2.3	1.73	1.3–2.7	0.069	1.3–2.7
FT_3_ (pmol/L)	4.4	3.0–6.2	5.3	3.9–9.3	0.005	3.3–8.2
TSH (mU/L)	1.70	0.04–4.50	0.70	0.01–2.03	0.001	0.5–5.00
TBII (U/L)	1.3	0.5–8.9	1.1	0.5–7.6	0.007	

Insulin (pmol/L)	30	15–131	32	15–136	0.695	34–172
Adrenalin (nmol/L)	0.08	0.05–0.48	0.09	0.05–0.51	0.872	0.00–0.55
Noradrenalin (nmol/L)	1.05	0.43–3.69	1.38	0.53–4.18	0.833	0.00–3.25

## Abbreviations

BRT: Block and replacement therapy
BW: Body weight
REE: Resting energy expenditure
TH: Thyroid hormone
MMI: Methimazole
PTU: Propylthiouracil
TSH-R: TSH receptor
TBII: TSH-R autoantibodies
rT_3_: Reverse T_3_
*V*O_2_: Oxygen consumption
*V*CO_2_: CO_2_ production
RQ: Respiratory quotient
RIA: Radioimmunoassay.
